# CD103 Deficiency Promotes Autism (ASD) and Attention-Deficit Hyperactivity Disorder (ADHD) Behavioral Spectra and Reduces Age-Related Cognitive Decline

**DOI:** 10.3389/fneur.2020.557269

**Published:** 2020-12-23

**Authors:** Michelle Jhun, Akanksha Panwar, Ryan Cordner, Dwain K. Irvin, Lucia Veiga, Nicole Yeager, Robert N. Pechnick, Hanna Schubloom, Keith L. Black, Christopher J. Wheeler

**Affiliations:** ^1^Department of Neurosurgery, Cedars-Sinai Medical Center, Maxine Dunitz Neurosurgical Institute, Los Angeles, CA, United States; ^2^Department Biomedical & Translational Sciences, Cedars-Sinai Medical Center, Los Angeles, CA, United States; ^3^StemVax Therapeutics, Chesterland, OH, United States; ^4^Department of Basic Medical Sciences, College of Osteopathic Medicine of the Pacific, Western University of Health Sciences, Pomona, CA, United States; ^5^Society for Brain Mapping & Therapeutics, Brain Mapping Foundation, Santa Monica, CA, United States; ^6^T-Neuro Pharma, Inc., Albuquerque, NM, United States

**Keywords:** autism, ADHD, CD103, CD8 T cell, cellular immunity, age-related cognitive decline

## Abstract

The incidence of autism spectrum disorders (ASD) and attention deficit hyperactivity disorder (ADHD), which frequently co-occur, are both rising. The causes of ASD and ADHD remain elusive, even as both appear to involve perturbation of the gut-brain-immune axis. CD103 is an integrin and E-cadherin receptor most prominently expressed on CD8 T cells that reside in gut, brain, and other tissues. CD103 deficiency is well-known to impair gut immunity and resident T cell function, but it's impact on neurodevelopmental disorders has not been examined. We show here that CD8 T cells influence neural progenitor cell function, and that CD103 modulates this impact both directly and potentially by controlling CD8 levels in brain. CD103 knockout (CD103KO) mice exhibited a variety of behavioral abnormalities, including superior cognitive performance coupled with repetitive behavior, aversion to novelty and social impairment in females, with hyperactivity with delayed learning in males. Brain protein markers in female and male CD103KOs coincided with known aspects of ASD and ADHD in humans, respectively. Surprisingly, CD103 deficiency also decreased age-related cognitive decline in both sexes, albeit by distinct means. Together, our findings reveal a novel role for CD103 in brain developmental function, and identify it as a unique factor linking ASD and ADHD etiology. Our data also introduce a new animal model of combined ASD and ADHD with associated cognitive benefits, and reveal potential therapeutic targets for these disorders and age-related cognitive decline.

## Introduction

A role for cellular immunity in neurodevelopmental disorders is suggested by prominent adaptive and innate immune abnormalities in autism, attention deficit, schizophrenia, and related conditions (1, 2). The specific contribution of adaptive cellular immunity to discrete neurodevelopmental conditions remains poorly defined, however, and discrete changes in cellular immune effectors have not been linked to particular neurodevelopmental disorder(s). Nevertheless, both autism spectrum disorders (ASD) and attention deficit hyperactivity disorder (ADHD) are associated with increases in adaptive immune (T) cell cytokines, specifically Th2-like cytokines (IL-4, IL-6, and/or IL-10), or decreases in Th2-like cytokines (IL-2 and/or IFNγ) (3, 4). In addition, the mucosal immune system has been particularly implicated in ASD, ADHD, and some learning disabilities (1, 2, 5–7).

The mucosal immune system is mediated by the action of particular subsets of innate and adaptive immune cells. Among the most prominent elements of the adaptive immune subcompartment are tissue-resident memory CD8 T cells derived from recent thymic emigrants that express the αE integrin (CD103) (8). In this context, CD103 is known to function as an E-cadherin receptor, facilitating T cell binding to epithelial cell surfaces and tissue entry across epithelial cell barriers such as gut and brain (8–10). Moreover, CD103 is in theory capable of modulating cadherin-mediated β-catenin activity, which critically regulates neuronal generation, function, and synaptic plasticity (11). In this context, T cell production and emigration to the general circulation coincides with critical milestones in higher brain development, including gliogenesis, synaptogenesis, and apoptosis (12, 13).

Autism spectrum and attention deficit disorders in particular result from abnormalities in the later stages of brain development such as socialization and executive functioning (1, 14). Moreover, these higher order brain functions are the first targets of neurodegeneration during aging and disease (15, 16). In theory, this makes autism, attention deficit disorder, and even neurodegeneration, particularly sensitive to the influence of tissue-resident and/or mucosal T cells. A direct impact of these cells on developmental signaling or behavioral consequences of late brain development is therefore possible but has yet to be demonstrated. This contrasts with the well-characterized cross-talk between central nervous and immune system signaling occurring via a variety of other mediators that include steroid hormones, cytokines, and those of the hypothalamic-pituitary-adrenal axis (17).

To examine this possibility, we validated the potential of CD103 expressed by CD8 T cells to modulate neural progenitor cell cycle entry and related signaling *in vitro*. We then monitored behavioral hallmarks of neurodevelopmental conditions in mice deficient for CD103, which are well-known to exhibit deficits in gut CD103^+^ immune cells (8, 18, 19). Following up on findings of autism spectrum- and attention deficit disorder-like behavioral characteristics in female and male CD103-deficient mice, respectively, we examined neuronal, synaptic, and transcriptional regulatory proteins in female and male CD103-deficient brains. Finally, we documented surprising protection from age-related cognitive loss in CD103-deficient mice generally. Our findings reveal a novel linked model of sex-disparate neurodevelopmental disorders from the same immunogenetic lesion, and suggest rationales for immune modulation in the treatment of ASD, ADHD, and age-related neurodegeneration.

## Materials and Methods

### Animal Subjects

Female C57BL/6, B6.Foxn1 mice, and syngeneic CD103-knockout strains (Jackson Labs) were housed in a pathogen–free vivarium under standard conditions on a 12-h light/12-h dark cycle with food and water *ad libitum*. Animals were administered behavioral tests at 8–10 weeks and 14 months old. Cell derivation from donor mice (co-cultures) was randomized by pooling from >5 donors per experiment. Young (8–10 wk) and aged (14 months) male and female C57BL/6 and B6.CD103-knockout mice (*n* = 12 young; *n* = 7–8 aged) were used to study age-related cognitive decline. All animals were maintained in a pathogen-free facility under the Cedars-Sinai Department of Comparative Medicine, with prior breeding and genetic screening conducted at Jackson Laboratories (Bar Harbor, ME).

### Tissue Processing (Brain, Spleen)

Brain and spleen was harvested from PBS-perfused mice. Brains were sectioned 1 mm to the right of the longitudinal fissure (midline). Right hemispheres were flash frozen in −80°C conditions for protein studies, followed by homogenization in Cell Lysis Buffer (Cell Signaling Technologies, MA), and centrifugation of nuclei. Cell lysates were separated into Triton soluble, Sarkosyl soluble and Sarkosyl insoluble fractions using sequential incubations of 10% (wt/V) salt sucrose solution and 1% (wt/v) sarkosyl Salt Sucrose Solution.

### Flow Cytometry

Purified T cells stained with indicated antibodies were analyzed by three-color flow cytometry (FACScan II; BD Biosciences, San Jose, CA) to assess purity. Antibodies were incubated with whole-spleen single cell suspension in PBS with 5% FBS, on ice for 30 min, followed by a wash with the PBS with 5% FBS. 100,000–300,000 flow events were acquired.

### Neural Progenitor Cell Isolation & Culture

C57BL/6J mice at E12, E18, and P0 were decapitated and brains removed from the cranium. Cortices were obtained and placed in calcium- and magnesium-free media. Meninges were removed and cells triturated with a fire-polished pipette. Cells were then spun at 600 g for 5 min and resuspended at 40,000 cells/ml in DMEM/F12 in the presence of 20 ng/ml bFGF (Sigma) or 50 ng/ml TGF-β (maximal concentration; Sigma), 2.5% penicillin/streptomycin (Gibco), and 1x B-27 (Gibco) and maintained at 37°C at 5% CO2. bFGF or TGF-∙ was added every 3 days and cells were passaged after 7 days. Single cell suspensions were generated by trituration of neurospheres in the presence of 0.05% Trypsin/EDTA for 15 min at 37°C and then pelleted at 600 g for 5 min. Trypsin/EDTA was removed and cells resuspended at low density (1,000 cells/ml) in 50% conditioned media/50% DMEM/F12 and passaged through a 20-μm mesh allowing for single cell isolation. Cells were then resuspended in DMEM/ F12 with 2.5% penicillin/streptomycin, and 1x B-27 and 20 ng/ml bFGF or 50 ng/ml TGF-∙ (maximal mitogenic concentrations). bFGF and TGF-∙ were added every day for up to 7 days. Cells were then collected and plated on poly-L-lysine-coated plates, in the presence of Neurobasal (Gibco) media with 5% penicillin/streptomycin, 200 mM L-glutamine, 25 mM L-glutamic acid and 1x B-27 and co-cultured with syngeneic C57BL/6J CD8 T cells.

### NPC-CD8 T Cell Co-culture

C57BL/6J CD8 T cells were purified by MACS anti-CD8 affinity column, and >90% purity assessed by flow cytometry. Neural Progenitor Cells (NPCs) were plated at a density of 50,000 cells/ml in proliferation media (DMEM/F12, 1X B-27, 5 ng/ml bFGF, 5 ng/ml EGF) on adhesive substrate, poly-ornithine/fibronectin, along with purified CD8 T-cells with or without simultaneous activation with 1 mg anti-CD3ε plus anti-CD28 mAb (for direct stimulation of T cell receptor, plus co-receptor stimulation, respectively), at 50,000 cells/ml. On the third day after initial plating, cells were sorted by FACS for Nestin (NPC marker) or CD8 (T cell marker) positivity, exposed to Propridium Iodide, and analyzed by flow cytometry for cell-cycle status.

### Western Blot

Triton-soluble cell lysates were electrophoretically separated on 12% Tris-HCl Precast Gels (Bio-Rad), and blotted onto 0.2 μm nitrocellulose. Membranes were blocked with BSA, incubated in sequential primary and secondary antibody dilutions for 1 h at room temperature with >3 washes, developed with enhanced chemiluminescence substrate (GE Healthcare Biosciences; Pittsburgh, PA), and exposed onto Amersham Hyperfilm (GE Healthcare Biosciences; Pittsburgh, PA).

### Antibodies for Cultures, Flow Cytometry and Westerns

Free-floating brain sections (8–14 μm thick) were mounted onto slides and blocked for 1 h at RT. Sections were incubated at 4°C overnight with primary antibody in blocking solution (Dako, CA). Sections were rinsed 4x in PBS, and incubated 90 min in fluorochrome- or biotin-conjugated secondary antibody. Sections were washed, coverslipped, and mounted with ProLongGold anti-fade media with DAPI (Invitrogen). Bright-field and fluorescent images were obtained using a Zeiss AxioImagerZ1 with CCD camera (Carl Zeiss Micro imaging). Image analysis of micrographs was performed with ImageJ (NIH). Anti-CD103 antibody (BD 550259) was used at 1:500 for flow cytometry (FC) 1:1000 for Western blot (WB), and 1 mg/ml for co-cultures. Anti-CD4 antibody (BD Pharmingen 2.43 for FC; Abcam ab133616 for WB) was used at 1:50 for FC and 1:100 for WB. Due to marker size and insuitability of GAPDH, WB signal was normalized to that of β-actin (control housekeeping protein; clone AC-74, Sigma). Anti-GAPDH (oxidative metabolism enzyme; Abcam ab9485) was used at 1:500 for WB. Anti-NeuN antibody (Neuronal progenitor marker; Chemicon) was used at 1:100 for WB. Anti-CD3ε (T cell receptor signaling protein; BD Pharmingen clone 145-2C11) and anti-CD28 (T cell co-stimulatory protein; BD Pharmingen clone CD28.2) were used at 1 mg/ml each for co-cultures. Anti-CD8 (BD Pharmingen clone 53-6.72, for FC; Abcam ab4055 for WB) was used at 1:100 for FC and 1:1000 for WB. Antibodies to the synaptic proteins, Synaptophysin (SY48; Abcam ab8049) and Drebrin (M2F6; Abcam ab12350), and anti-eIF4E (translation initiation factor implicated in ASD; Y449; Abcam ab33768), were all used at 1:500 for WB. All secondary antibodies (HRP, Alexa Flour-488,−594,−647; Invitrogen) were used at 1:200 for IHC and 1:2000 for WB.

### Behavioral Testing

The Open Field test was performed preceding other behavioral tests, at 2.5–3 months and again at 14 months of age. Barnes Maze testing was performed at 3 and 14 months of age. The order of behavioral tests was randomized by alternating control and treatment group animal runs. Tests were begun at the same time (+/−1.5 h) for tests run on more than 1 day, with early and late times alternated for inter-group randomization. For the Barnes maze, additional randomization of alternating escape compartment location between each animal per group, and between each of 3 daily training tests per animal, was employed.

#### Repetitive Grooming Test

Individual mice of each strain were first individually acclimated in a video-equipped cage for 10 min. Number of grooming sessions (>10 s between sessions) and total seconds spent grooming were determined by video surveillance at 10, 15, and 20 min after acclimation, in three sequential, 5-min recording sessions. CD103-deficient and wild-type C57BL/6 mice were randomly distributed during each testing run, with females and males tested on different days. Intra-strain and -sex grooming session numbers and total time spent grooming were not significantly different between any of the three recording sessions, and hence their metrics were combined by strain and sex exclusively.

#### Y-Maze Spontaneous Alternation (SA)

Y-Maze Alternation Test is used to assess working memory. Spontaneous alternation was measured by individually placing animals in one arm of a symmetric Y-maze made of opaque black acrylic (arms: 40 cm long, 4 cm wide; walls: 30 cm tall), and the sequence of arm entries and total number of entries recorded over a period of 8 min. Mice were tested for SA a single time only.

#### Barnes Maze (BM)

Barnes maze is a spatial-learning task that allows subjects to use spatial cues to locate a means of escape from a mildly aversive environment (i.e., the mice are required to use spatial cues to find an escape location). Mice were assessed for their ability to learn the location of an escape box over the course of 9 days in the BM apparatus. The escape hole is constant for each mouse over the five training days. Each mouse was tested three times per day (3 trials) for 4 days, followed by no testing for 2 days, and re-testing on day 7. A 35–60 min inter-trial interval separates each trial. Each trial began by placing one mouse inside a start box with a bottomless cube positioned centrally on the maze. After 30 s, the start box was lifted and the mouse was released from the start box to find the escape hole. Two fluorescent lights located on the ceiling or high above illuminate the testing room. Each trial lasted up to 4 min or until the mouse entered the escape box. The experimenter guided mice that failed to find the escape hole within 4 min, to the correct hole after each training test. Once the mouse entered the escape box, it was allowed to remain in the box for 1 min. Following the 7th day of testing, and never on the same day, mice were tested an additional 2-days, in which the escape box was placed in the reverse position on day 8, and replaced in the original position on day 9. The same exact testing procedure was applied to all mice in all groups. The maze and all compartments were cleaned thoroughly with isopropyl alcohol to remove any olfactory cues after each trial, and prior to each day of testing.

#### Open Field Test

The test was carried out in an Open Field apparatus made up of an open topped, clear Plexiglas box, measuring “16 x 16” and 15” high. Two rings of photobeams and optical sensors surrounded the box. The optical sensors were connected to a computer by way of an input matrix. Each mouse was placed into the box, and breaks in the beam interruptions automatically recorded and used as a measure of locomotor activity. Each mouse was tested in the box for a period of 30 min.

#### Social Approach Test

The Social Approach test (SAT) has been used to detect behavioral correlates of autism in mouse models (20, 21). The SAT apparatus consists of a 3 chamber system made of clear Plexiglas measuring ~20 × 40 cm per chamber. The test was conducted in 3 phases. In the first phase, the 3 chambers were separated with solid dividers. A subject mouse was placed in the center chamber and allowed to habituation to the apparatus for 5 min. After 5 min, a mouse unfamiliar to the subject mouse was placed into a small metal cage and placed into 1 of the two side chambers. An identical empty cage was placed into the adjacent side chamber and the dividers were removed. In phase 2, the subject mouse was allowed to explore all 3 chambers freely for 10 min. The duration of time that the subject mouse spent in the chamber containing the unfamiliar mouse and the duration of time it spends oriented toward the cage with its nose < ~2 cm from it is recorded by a trained observer blind to treatment condition. In phase 3, another unfamiliar mouse was placed into the previously empty cage. The subject mouse freely explored the apparatus for 10 min. The duration of time spent in contact or oriented toward the new cage containing the unfamiliar mouse compared to the cage with the original unfamiliar mouse was recorded by a trained observer blind to treatment condition. For internal consistency the unfamiliar stimulus mice were matched by strain, genotype, and sex.

#### Statistical Analysis

Quantification of area was analyzed in six to eight coronal sections from each individual, at 150-μm intervals (unless otherwise indicated), covering 900–1,200 μm of forebrain areas. Specific marker signal was captured with the same exposure time for each image and sections from each field of the specimen were imported into NIH Image J and analyzed as above. GraphPad Prism (version 5.0b; San Diego, CA, USA) was used to analyze the data using ANOVA and *T*-Tests with Welch's correction (no assumption of equal variance). In all histograms, average ± SEM is depicted. Pre-determined exclusions included sections or samples with no discernible background signal, and values within each group >2 standard deviations above or below the median/group.

### Study Approval

All animal procedures were approved prior to performance by the Cedars-Sinai Institutional Animal Care and Use Committee.

## Results

### CD8 T Cells and CD103 Modulate NPC Proliferation and β-Catenin Signaling

**Figure 1:**
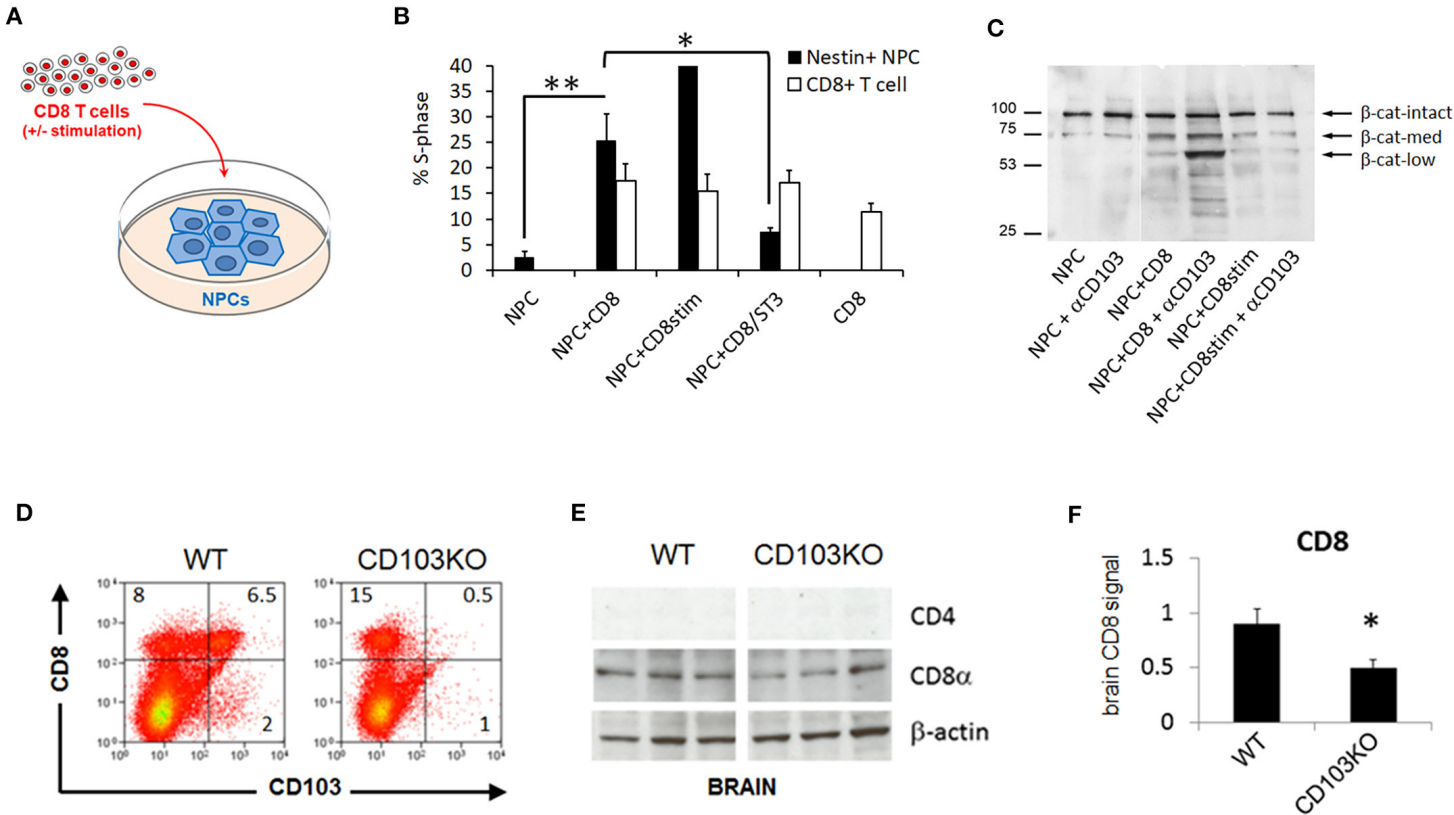
CD103 modulates CD8 T cell-mediated neural progenitor cell status and CD8 content in brain. Neural progenitor cell (NPC) + CD8 T cell co-culture schematic **(A)**; Percent S-phase of post-culture sorted NeuN^+^ NPCs but not CD8 T cells is significantly increased by co-culture **(B)**; β-catenin cleavage products are increased in post-culture sorted NeuN^+^ NPCs by co-culture with unstimulated CD8 T cells, and further enhanced by addition of anti-CD103 mAb to co-cultures **(C)**. CD103-deficient mice (CD103KO) exhibit expected decrease in splenic CD8 T cells **(D)** as well as brain CD8 **(E,F)** relative to age- and sex-matched wild-type C57BL/6 controls (WT). Co-culture data represent results compiled from 4 biological replicates; flow cytometry are representative of >4 independent mice per strain; Western data are representative of 2 independent experiments. *P < 0.05, **P < 0.01, by 2-sided *T*-Test in >4 biological replicates per sex per strain (normal distribution/*P* < 0.05 of data confirmed in Shapiro-Wilk test).

We initially combined cultured neural progenitor cells (NPCs) with CD8 T cells in overnight co-cultures and assessed cell cycle status in each cell population by flow cytometry (Figure 1A). While cell cycle entry of CD8 T cells was not significantly impacted by NPCs in such co-cultures, NPC cell cycle entry was increased nearly 10-fold by CD8 T cells (Figure 1B). In addition, pre-treatment with ST3Gal-II, a sialotransferase we showed preferentially impairs CD103^+^ CD8 T cell function (22) (Supplementary Figure 1), eliminated the CD8 T cell-mediated increase in S-phase NPC (Figure 1B). To explore a possible mechanism for this, we examined cleavage in NPCs of β-catenin, a downstream proliferation-modulating signal of the CD103 ligand E-cadherin, upon co-culture with CD8 T cells in the presence or absence of an anti-CD103 antibody (M290) capable of either blocking CD103 ligation or costimulating CD103^+^ T cells (23). As expected, addition of CD8 T cells induced increased β-catenin cleavage in NPCs (Figure 1C). More surprisingly, addition of anti-CD103 antibody, while inconsequential by itself, induced much more marked β-catenin cleavage when combined with CD8 T cells in co-culture (Figure 1C), notably resulting in a prominent low molecular weight β-catenin species associated with cell proliferation in inflamed and cancerous cells (24). Although it is formally possible that blocking CD103 ligation with the M290 antibody is responsible for the observed effects on NPC proliferation, we consider its co-stimulatory activity on CD103^+^ T cells a more likely explanation when considered together with cell cycle data. Alternatively, the low molecular weight β-catenin species seen on Westerns with M290 could mediate non-proliferative activities in cultured NPCs. In either case, our findings demonstrated CD103 involvement in CD8 T cell-mediated modulation of neurodevelopmental cell function, and as such justified examining neurodevelopmental behavioral consequences of CD103 deficiency in general.

### CD103-Deficiency Decreases CD8 Signal in Brain

CD103 deficiency is known to primarily affect subpopulations of T cells, including intraepithelial lymphocyte T cells and peripheral regulatory CD4 and CD8 T cells, but also a subset of dendritic cells in the gut mucosa and mesenteric lymph nodes (8, 25), We confirmed that CD8 T cells were the most prominent peripheral T cell population impacted in CD103-deficient mice (Figure 1D). CD103^+^ CD8 but not CD4 T cells are also known to populate mouse brain parenchyma (9, 10, 26). Our previous work indicated that counting CD8 T cells in disrupted brain tissue, either by flow cytometry or by immunofluorescence, resulted in high variability and low absolute numbers in our hands, consistent with the known propensity of resident-memory CD8 T cells in brain to die upon tissue disruption (26–28). In contrast, Western blot more accurately quantified independently established differences (i.e., age-related accumulation) in brain CD8 T cells (29, 30). We thus concluded that detection methods that do not rely on intact cell structure are superior to tissue staining for quantifying these cells in brain. Accordingly, we used Western blot to detect CD8 and verify the absence of CD4 signal in wild-type as well as CD103-deficient forebrain (Figures 1E,F). CD8 signal in CD103-deficient brain was about half that in wild-type (Figures 1E,F), consistent with the known ability of CD103 to facilitate CD8 T cell entry into brain (9, 10, 26). Notably, CD8 signal was reduced in both female and male CD103-deficient forebrain, but only reached significance in females (data not shown; 1.52 ± 0.64 WT vs. 0.496 ± 0.071 KO, *P* = 0.1584 in males; 1.284 ± 0.1 WT vs. 1.127 ± 0.087 KO, *P* = 0.0014 in females). Although this does not rule out an impact on other CD103^+^ immune cell subpopulations in brain, or alternative mechanisms such as greater protease sensitivity in CD103KO cells of any lineage, the reduction of CD8 signal in KO host brain is most consistent with the known ability of CD103 to facilitate brain entry/retention of the most prevalent CD103^+^, CD8 T cells.

### CD103-Deficiency Promotes Hyperactivity and Learning Deficit in Males

Given the potential impact of CD103 on both immune and neural cells relevant to neurodevelopment, we examined behaviors associated with neurodevelopmental differences in CD103-deficient relative to wild-type mice to determine if the absence of CD103 conferred neurodevelopmental consequences. Behavioral tests included Open Field, for movement; Y-Maze/Spontaneous Alternation for novelty preference and/or learning and memory deficits; Self-Grooming for repetitive behavior differences; Novel Partner Preference for socialization differences; and Barnes Maze for learning and memory alone. Because neurodevelopmental differences can exhibit sex differences, all tests were conducted in females and males separately.

**Figure 2:**
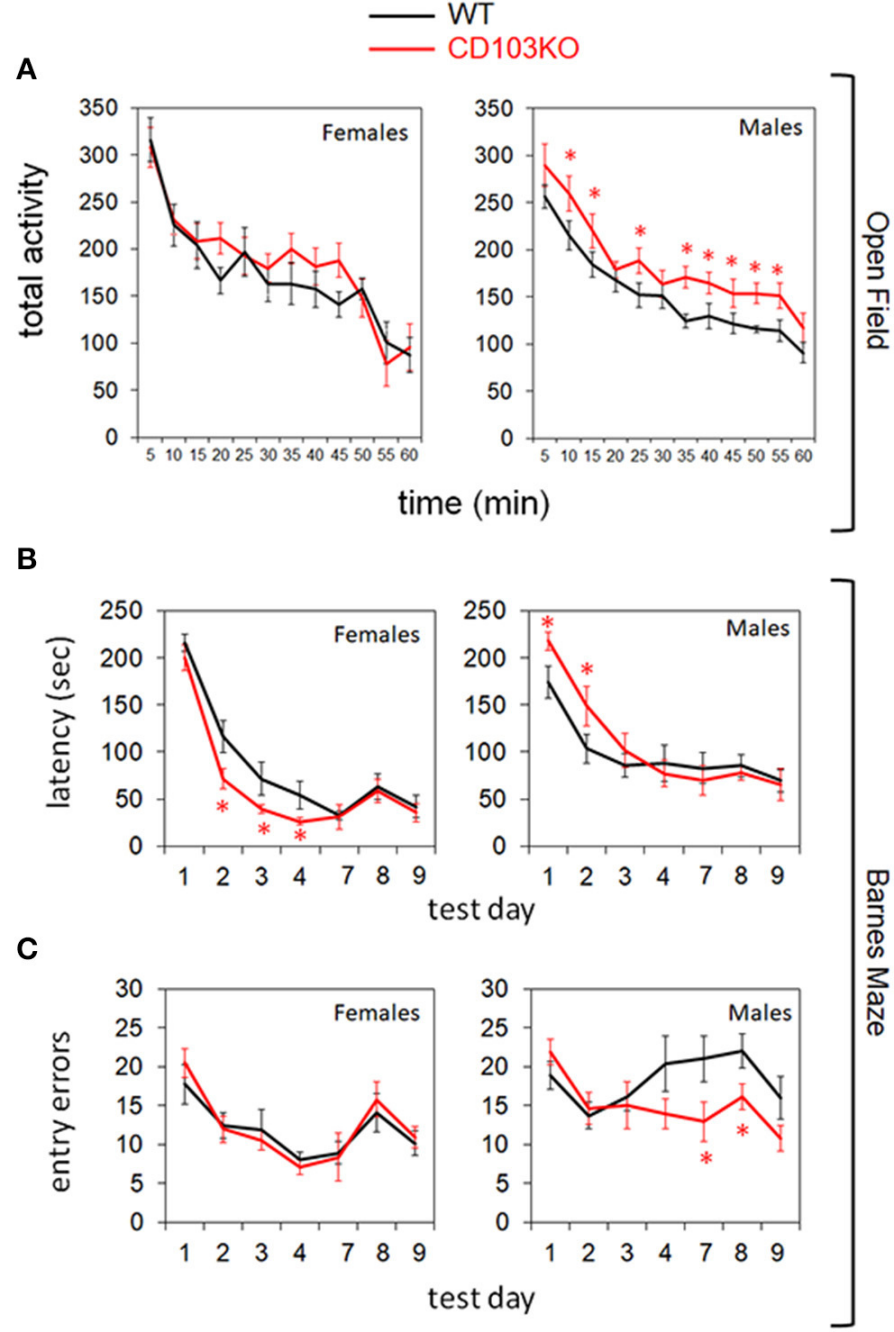
CD103-deficiency results in distinct movement and learning profiles in females and males. Open Field testing revealed overt hyperactivity in CD103-deficient (CD103KO) males exclusively **(A)**, while Barnes Maze testing revealed initially slower learning in male CD103KOs, but intrinsically superior learning in female CD103KOs **(B)**. Male CD103KOs eventually completed the maze as successfully as WT counterparts or female CD103KOs, in part because they committed fewer entry errors than either with training **(C)**. Open Field testing was performed on 11 mice per sex per strain; Barnes Maze was performed on >10 mice per sex per strain. *P < 0.05 by 2-sided *T*-Test for individual test points; *P* < 0.005 for female and male CD103KO vs. WT latency test curves, and for CD103 vs. WT male entry error test curves, by ANOVA (normal distribution/*P* > 0.05 of data verified in Anderson-Darling, D'Agostino & Pearson, Shapiro-Wilk, and Kolmogorov-Smirnov tests).

While no consistent movement difference was observed in Open Field by young (8–10 wk) CD103-deficient females, the males exhibited consistent hyperactivity relative to wild-type (Figure 2A). While this was expected to translate to superior performance of CD103-deficient males in subsequent time-dependent tests such as the Barnes Maze, this was not the case (Figures 2B,C). Indeed, CD103-deficient females not only outperformed their male counterparts for Barnes Maze latency, they also outperformed female wild-types during the training phase of the test (Figure 2B). Female CD103-deficient mice were equivalent to their wild-type counterparts after the 4-day training phase (Figure 2B), and in errors committed throughout the test (Figure 2C). By contrast, male CD103-deficient mice were slower than wild-type males in learning portions of the Barnes Maze (Figure 2B), but caught up after training, where they committed fewer errors than wild-type males (Figure 2C). Thus, CD103-deficent females were faster learners, and the males hyperactive with initially slower but trainable learning, compared to wild-type mice.

### CD103-Deficiency Promotes Distinct ASD- and ADHD-Like Behaviors in Females and Males

#### Spontaneous Alternation

**Figure 3:**
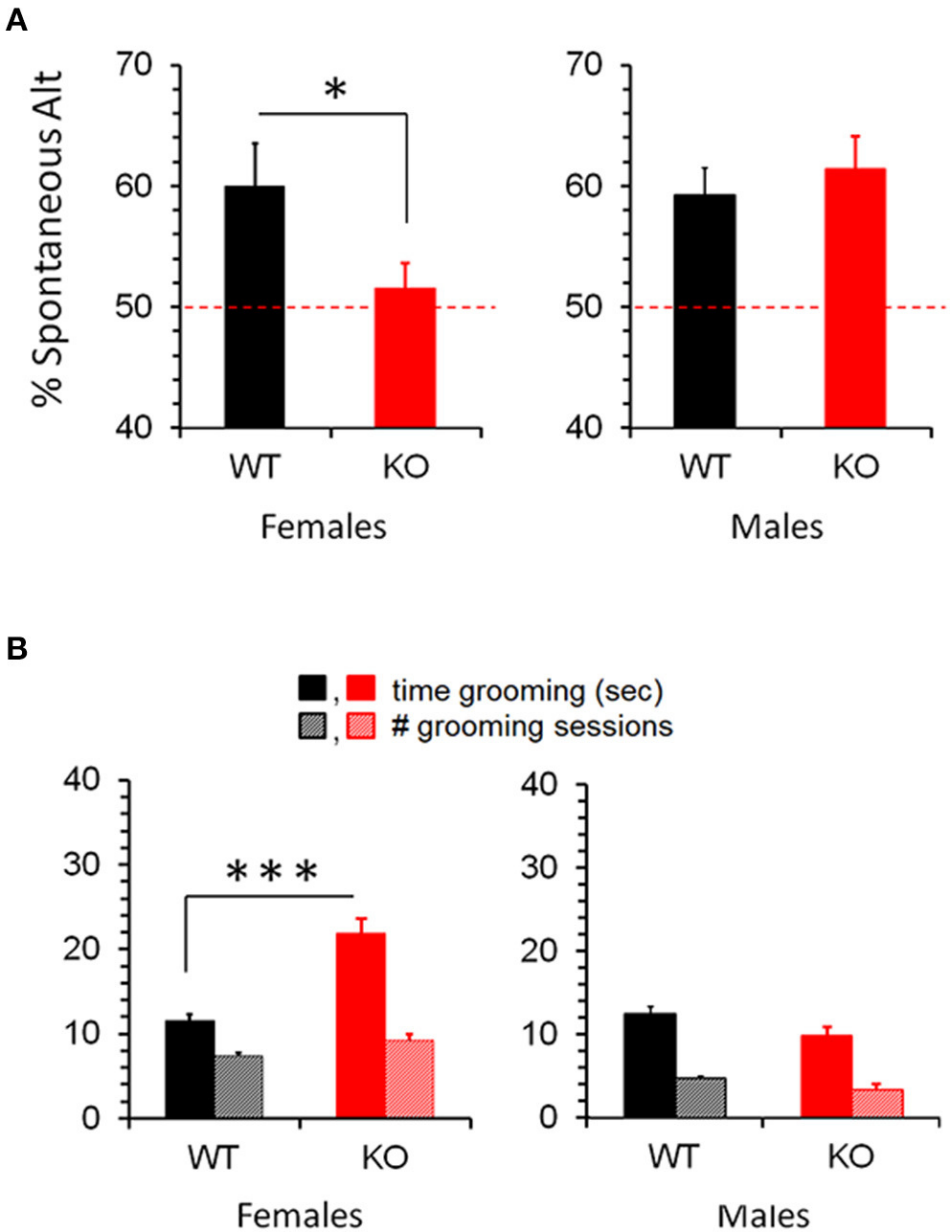
Increased Spontaneous Alternation and Repetitive Grooming in CD103KO females. Y-maze revealed absence (50%) of Spontaneous Alternation **(A)**, and markedly longer repetitive grooming **(B)** in CD103-deficient (KO) females exclusively. Tests were performed on 12 individual mice per strain per sex, with a single individual female per strain excluded as a statistical outlier (< 2 standard deviations below average). *P < 0.05 by 1-sided Mann Whitney test for Y-maze/Spontaneous Alternation (due to non-normal distribution/*P* < 0.05 of CD103KO data in Anderson-Darling, Shapiro-Wilk, and Kolmogorov-Smirnov tests); ***P < 0.005 by 2-sided *T*-Test for Grooming time (normal distribution/*P* > 0.05 of data verified in Anderson-Darling, D'Agostino & Pearson, Shapiro-Wilk, and Kolmogorov-Smirnov tests).

The Y-maze quantifies the tendency of a mouse to choose a distinct path from the one it previously chose (deemed Spontaneous Alternation), and hence requires memory of its previous choice. It can thus be seen as a rough test for learning/memory, but is not as definitive as the Barnes Maze and other more specialized learning/memory tests. If learning/memory deficits are ruled out, for example, Y-maze measures intrinsic preference for novelty. Given these considerations, Y-maze might be expected to parallel the differential learning seen in CD103-deficient females and males. On the contrary, however, CD103-deficient females exhibited a significant reduction to random chance (50%) in Spontaneous Alternation in the Y-maze relative to wild-type females, whereas CD103-deficient males exhibited no such reduction relative to wild-type (Figure 3A). In light of their superior learning/memory in the Barnes Maze, this suggests that CD103-deficient females are averse to novelty, whereas the learning deficit seen in the Barnes Maze by CD103-deficient males did not appreciably impact Spontaneous Alternation.

#### Self-Grooming & Socialization

**Figure 4:**
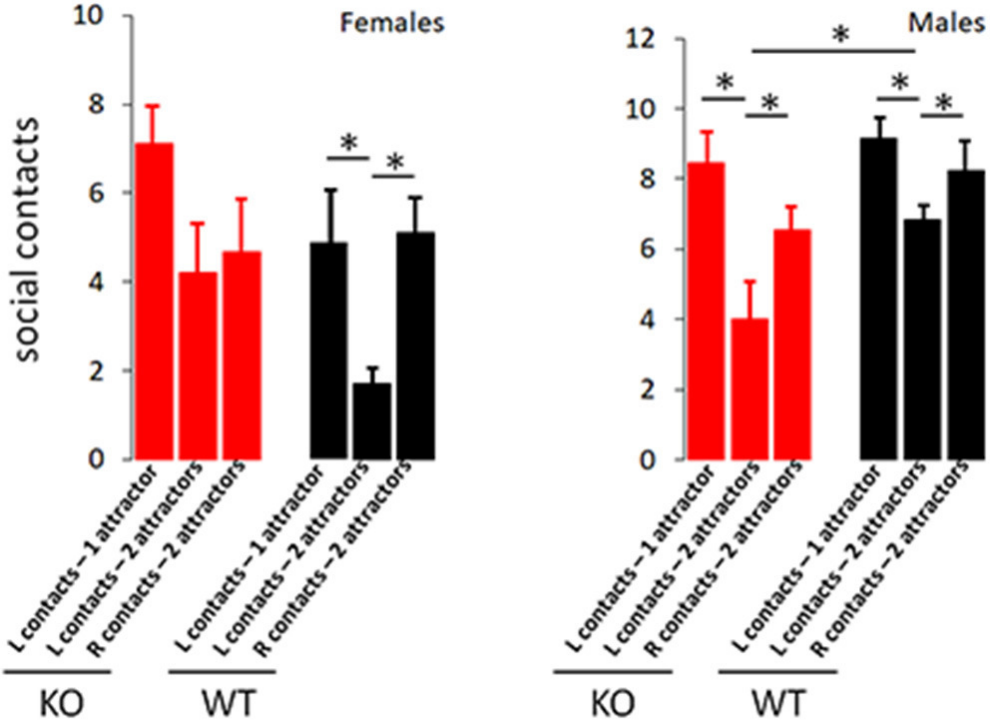
CD103-deficiency selectively alters socialization in females. Socialization test revealed expected preferential interaction with novel vs. familiar attractors in wild-type males and females, and male CD103-deficient. Female CD103-deficient mice exhibited no preference for novel vs. familiar, while CD103-deficient mice engaged in significantly fewer social contacts than wild-type with initial attractors. L contacts = contacts with initial (familiar) attractor; R contacts = contacts with 2nd (novel) attractor. Test was performed on >11 individual mice per sex per strain. *P < 0.05 by 2-sided *T*-Test in >9 individual mice per sex per strain, or in Mann-Whitney test (where non-normal distribution/*P* < 0.05 was verified by Anderson-Darling, D'Agostino & Pearson, and Shapiro-Wilk, tests, i.e., in comparisons with male L contacts; 2 attractors).

Self-Grooming timing and incidence are classical metrics for repetitive behavior. While incidence of self-grooming episodes was not impacted by CD103-deficiency in either females or males, females spent about twice as long grooming as either male CD103-deficient or wild-types of either sex (Figure 3B). CD103-deficient females also showed equal preference for interacting with familiar and novel partners, which is opposite the trend seen in CD103-deficient males or wild-types of either sex, all of which spent more time interacting with novel partners – the normal pattern of socialization in mice (Figure 4). Male CD103-deficient mice did, however, tend to spend slightly more time interacting with familiar partners than did their wild-type counterparts (Figure 4). Thus, female CD103-deficient mice are superior learners, averse to novelty, more prone to at least one type of repetitive behavior, and socially impaired. Male CD103-deficient mice, by contrast, are hyperactive, are slow but unique learners, have normal preference for novelty and no increase in repetitive behavior, and socialization that differs subtly from normal but is generally unimpaired. These behavioral characteristic are grossly similar to autism spectrum disorder (ASD) in the females, and attention deficit hyperactivity disorder (ADHD) in the male CD103-deficient mice.

### CD103-Deficient Mice Exhibit Disorder-Specific Brain Protein Modulation

**Figure 5:**
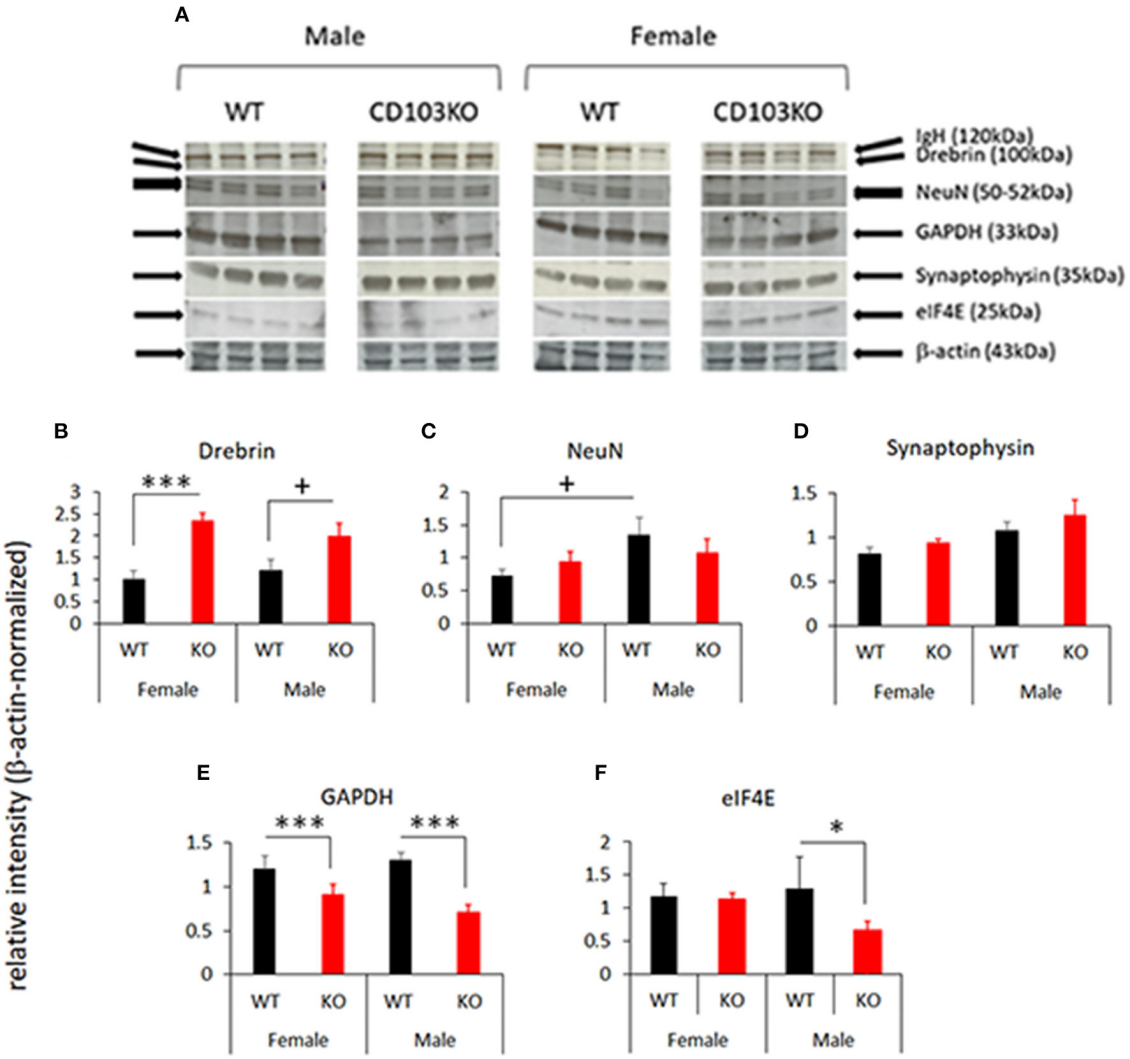
Selected protein markers in female and male CD103-deficient brain. Female and male forebrain from WT and CD103KO mice was homogenized and lysed in detergent, run on SDS PAGE gels, blotted and probed with the indicated antibodies **(A)**. Relative signal was normalized to β-actin in the same gel/blot lane, and results compiled from >4 animals per sex per strain. ^+^*P* < 0.1 by 1-sided Mann Whitney test for male Drebrin (due to non-normal distribution/*P* < 0.05 in Shapiro-Wilk test; **B**); *P < 0.05, ***P < 0.005 by 2- sided *T*-Test for NeuN **(C)**, Synaptophysin **(D)**, GAPDH **(E)**, and eIF4E **(F)**, which all had normal distribution/*P* > 0.05 of data verified in Shapiro-Wilk test.

ASD and ADHD are each characterized by neuropathological features that can be monitored by selected protein biomarkers. Human ASD, for example, exhibits increased synaptic density that can be reflected in increased synaptic relative to neuronal markers (31). In addition, increased oxidative stress in brain is characteristic of both ASD and ADHD in patients (32, 33), and can be reflected by down-regulation of oxidative metabolic proteins such as GAPDH (34). Moreover, overexpression of transcriptional regulatory proteins such as eIF4E are sufficient to promote verified ASD-like behaviors in mice (35, 36). To ensure correspondence of behaviors seen in CD103KO mice to human ASD and/or ADHD, as well as to identify possible mechanistic targets to modulate such behaviors, we therefore quantified NeuN, Drebrin, Synaptophysin, GAPDH, and eIF4E in separate female and male wt and CD103 cohorts. Both female and male CD103-deficient brains exhibited markedly decreased GAPDH with equal loading of cell lysates (Figures 5A,E). Since this protein is commonly used as a protein-loading control on Western blots, we used β-actin for protein controls instead (along with incidental IgH from secondary reagents for additional verification). The decrease in GAPDH in both female and male CD103-deficient brains reflects oxidative stress, and is consistent with this shared feature of ASD and ADHD. Drebrin (a synaptic protein), was also generally increased in CD103-deficient brains, although significantly so only in females (Figures 5A,B). No significant change was seen in NeuN (Figures 5A,C), nor in the synaptic protein, Synaptophysin, though the latter was incrementally increased in CD103-deficient brains (Figures 5A,D). The increased ratio of Drebrin to NeuN in females, however, suggests an increase in synaptic density exclusively in CD103-deficient females (Figures 5B,C), consistent with ASD neuropathology. Finally, levels of eIF4E, were significantly greater in CD103-deficient females than males, consistent with female-restricted ASD. Nevertheless, eIF4E was actually decreased in CD103-deficient males relative to wt (Figures 5A,F), revealing the possibility that its reduced expression may confer resistance to ASD-like behavioral symptoms. Thus, markers of brain oxidative stress and increased synaptic density validate correspondence of behaviors in CD103-deficient mice to human disorders, while the reduction in eIF4E suggests a potential molecular mechanism involved in sex-specific behavioral differences in CD103-deficent mice (i.e., reduced expression of an ASD-promoting protein precluding ASD-like symptoms in males).

### CD103 Deficiency Reduces Age-Related Cognitive Decline in Males and Females

Human ASD has been reportedly associated with protection from some aspects of age-related cognitive loss (37, 38), although this linkage has not been established in experimental models of ASD. Moreover, CD103^+^ lymphocytes and CD8 T cells in particular accumulate in mouse and human brain with aging (30). We therefore examined cognitive decline in aging CD103-deficient mice to further verify the model's correspondence to human-specific features, as well as to determine if CD103 on immune cells represents a potential target to improve age-related cognitive decline. Aging CD103-deficient females developed severe cutaneous skin lesions due to repetitive scratching more frequently than males, although the difference in our small colony was not statistically significant. This ultimately led to veterinary staff mandating early euthanasia in more females than males. Due to this unequal mortality between sexes, we performed combined analysis of age-related cognitive decline in both sexes of CD103-deficient mice, with matched sex ratios in wild-type controls. Increased errors in the Barnes Maze is a prominent age-related change on the C57BL/6 genetic background (39). We thus examined this and other parameters of the Barnes Maze in 14 month-old (“aged”) CD103-deficient and wild-type (C57BL/6) mice, after Open Field testing for locomotion differences.

**Figure 6:**
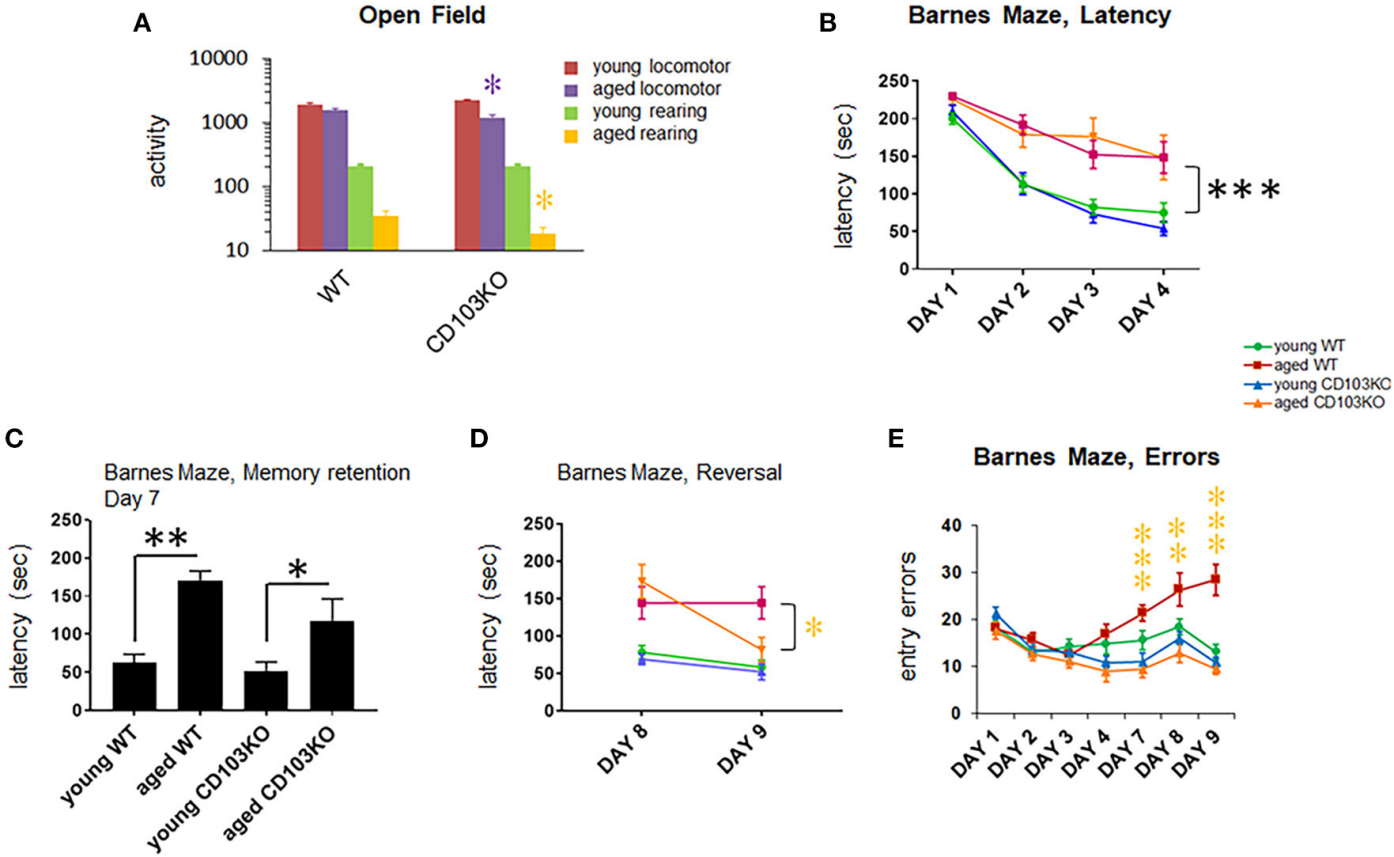
CD103 deficiency reduces age-related cognitive decline in males and females. All aged (14 mos old) mice exhibited substantially reduced locomotion and rearing relative to young (10 wk old) mice (statistics not shown; *P* < 0.01). Aged CD103KO mice also exhibited significantly decreased locomotion and rearing in Open Field than wild-type mice **(A)**. Barnes Maze testing revealed equivalent latency in aged CD103KO relative to aged WT during training [days 1–4; **(B)**] and memory retention phases of the test [day 7; **(C)**]. At reversal phases (day 8, 9) aged CD103KOs surprisingly exhibited latency similar to young mice irrespective of strain, whereas aged wild-type had significantly higher latency **(D)**. Aged CD103KOs also exhibited markedly reduced entry errors than aged wild-type at both memory retention and reversal phases of the test **(E)**. WT and CD103KO groups contained equal proportions of females and males (*n* = 3 and 7, respectively). *P < 0.05, **P < 0.01, ***P < 0.005 by 2-sided *T*-Test for individual test points (normal distribution/*P* > 0.05 of data verified in Anderson-Darling, D'Agostino & Pearson, Shapiro-Wilk, and Kolmogorov-Smirnov tests).

Aged CD103-deficient mice exhibited significantly reduced overall activity and rearing in Open Field, leading to our expectation that they would perform slower in Barnes Maze (Figure 6A). Remarkably, however, CD103-deficient mice exhibited maze latency times similar to age-matched wild-type counterparts in the training and memory retention phases (days 1–4, and day 7, respectively), although aged mice of either strain were consistently slower than younger counterparts (Figures 6B,C). In the last reversal phase of the test (days 9), however, aged CD103-deficient mice exhibited significantly faster latency than aged wild-type, which was similar to that of young mice of either strain (Figure 6D). Error analysis also revealed that CD103-deficient mice committed progressively fewer entry errors at later test phases (Figure 6E). Overall, this indicates that aged CD103-deficient mice are protected from prominent aspects of age-related cognitive decline revealed in the Barnes Maze, while they appear somewhat more sensitive to age-related changes in activity. Behavioral data in CD103-deficient females and males is compiled in Table 1.

**Table 1:** Behavioral summary of CD103KO mice by age and sex.

**Test → Age/sex ↓**	**Locomotor activity (open field)**	**Repetitive grooming**	**SA/novelty preference**	**Socialization**	**Barnes maze latency**	**Barnes maze errors**
Young female	Normal	Increased	Reduced	Reduced	Faster than wt +/– training	Normal
Young male	Increased (hyperactive)	Normal	Normal	Normal	Slower than wt until trained	Fewer than wt after training
Aged female	ND	ND	ND	ND	Faster than wt after training	Normal
Aged male	ND	ND	ND	ND	Normal	Fewer than wt after training

*All comparisons are relative to age- and sex-matched wild-type (wt) C57BL/6 cohorts*.

## Discussion

Several animal models for ASD and ADHD exist, including socially deficient rodent strains, induction of behavioral symptoms by drugs or severe maternal inflammation, and most prominently for ASD, transgenic mice expressing mutations associated with the human condition (40). These models often exhibit behavioral abnormalities with or without supportive neuropathology. Rodent models of ASD and ADHD do not consistently reproduce basic demographic hallmarks such as sex disparity and linkage to co-morbidities (41–43), however, and more subtle properties in patients such as altered age-related cognition (37, 38) or accompanying savant skills (40, 44–46). Moreover, no model provides a biological basis for the frequent co-occurrence of ASD and ADHD, although their etiological linkage has been hypothesized in humans (47–50), and were recently found to share similar gene mutations (51). Thus, models that better reflect the human conditions separately and coordinately are needed.

Both ASD and ADHD are influenced by environmental, metabolic, genetic and immune factors. With regard to genetic factors, mutations in several developmental signaling genes are associated with rare cases of ASD in particular. For example, deletion of the gene encoding β-catenin (CTNNB1) in neurons was recently found to cause ASD-like behavior (52), and rare mutations have been identified in patients with learning defects and autism spectrum disorder (ASD) (53, 54). β-catenin is a key developmental signaling protein that regulates cell differentiation and proliferation and is itself regulated by extracellular cadherin adhesion (55).

Similarly, among immune factors, pro-inflammatory cytokines such as IFNγ that are capable of regulating neurodevelopmental processes as well as key neuroinflammatory cells such as microglia, are also typically upregulated in ASD and ADHD (3, 6, 56). Prominent changes in innate immune and resident memory T cells (T_RM_) in gut also occur in ASD. These cells frequently express the αE-integrin, CD103, a known cadherin receptor that is at least in theory capable of modulating neurodevelopmental signaling through β-catenin (8, 18, 57). Nevertheless, identifying properties linking the diverse risk factors in ASD/ADHD has been elusive.

A low level of CD8 T_RM_ cells exist in brain parenchyma after birth, while other adaptive immune cells such as B and CD4 T cells are excluded (26, 29, 58, 59). We and others have shown that these brain CD8 T cells are also prominently CD103^+^, and that they accumulate with advancing age (9, 10, 26, 29, 30). This is intriguing, because ASD in particular has been linked to distinct age-related cognitive trajectories in some human studies (37, 38). Nevertheless, the impact of CD103^+^ immune cells on developmental signaling, neurodevelopmental disorders, and age-related behavioral changes has not been previously explored.

In this study, we first established in mixed co-cultures that CD8 T cells in particular can modulate β-catenin signaling in neural progenitor cells (NPC), and that CD103 was required for both maximal modulation and its functional outcomes on NPC. While we were not able to determine the precise molecular nature of CD103-mediated signaling in neural progenitors upstream of β-catenin, its involvement was sufficient to encourage examination of the behavioral consequences of CD103 deficiency in knockout mice, particularly after verifying that CD8 T cells were the most prominent immune cell subpopulation in such mice.

We then documented that CD103 deficiency results in behavioral changes consistent with neurodevelopmental disorders, specifically ASD and ADHD, in knockout females and males, respectively. Specifically, CD103 deficiency significantly reduced CD8 levels in brain, consistent with a possible impact of CD103^+^ CD8 T cells on brain function, and consistent with the known role of CD103 in homing of CD8 T cells to epithelial tissues including brain (8–10). Remarkably, CD103-deficient females exhibited normal motor activity, but deficient socialization, aversion to novelty, and increased repetitive behavior, without apparent cognitive deficits. Indeed, CD103-deficient females consistently outperformed normal female counterparts in hippocampus-dependent maze performance. By contrast, CD103-deficient males exhibited overt hyperactivity and delayed learning/memory, but only very minor social deficit, no aversion to novelty, and no increase in repetitive behavior. Moreover, CD103-deficient females and males were partially protected from age-related cognitive decline, albeit in distinct ways. These behavioral differences were paralleled by distinctly altered content of neuronal, synaptic, and transcriptional regulatory proteins in female and male CD103-deficient brains.

Taken together, these findings suggest that a singular immune defect in CD103 deficiency promotes ASD-like symptoms and neuropathology in females, while promoting ADHD-like symptoms and neuropathology in males. These symptoms were associated with potentially superior cognitive abilities, as female Barnes Maze performance was superior to wild-type even without training, and males committed fewer errors in the maze after training, and both were protected against some aspects of cognitive decline with aging. This beneficial component is particularly intriguing, given reports that high-functioning forms of ASD such as Asperger's Syndrome can be associated with super-normal ability in focused areas (40, 44–46). Our model is thus unique in integrating multiple disease properties from a single inductive lesion, and as such may offer superior mechanist insights into ASD, ADHD and their linkage.

Limitations of our findings include that mice were not tested for impaired vocalization, or for a fuller battery of tests to more fully characterize behavioral profiles. Vocalization is typically recorded by male pups to mothers, and since males did not exhibit other behaviors consistent with ASD, we chose not to pursue this. Additional tests for repetitive behavior and cognition, such as nesting and novel object recognition, in addition to alternative tests such as Morris Water Maze, will be important components to further validate our model's relevance to human disorders. A further limitation is that the mechanistic details of immune-neural signaling were not elaborated at the cellular or molecular level in our study. In this context, the contribution of CD8 T vs. other CD103^+^ innate and adaptive immune cell subpopulations, as well as the signaling pathways induced by CD103 ligation impacting β-catenin, require more detailed analysis in future studies. A final limitation is that the sex disparity for ASD- and ADHD-like behaviors in our model is the opposite of that seen in humans: ASD is more prevalent in human males by a factor of 4 to 1, while sex differences in ADHD are more complicated (60). It's difficult to see how such a distinct difference in sex disparity could be ascribed to hormonal differences in the two species, but this needs to be formally addressed. If ruled out, however, examination of factors with opposite sex linkage in mice and humans may be warranted. Indeed, cadherin-like proteins may be among such factors (61), though their relationship to CD103^+^ immune cells has not been examined.

Since CD103 is known to direct homing of immune cells expressing it to gut in addition to brain (8–10), our findings additionally suggest that this immune factor may link brain-gut-immune dynamics in ASD and ADHD. Moreover, it lends mechanistic credence to popular treatments for ASD and ADHD based on gut-brain-immune modulation (6, 56, 62, 63). It is thus tempting to speculate that directly targeting CD103 and/or immune cells expressing it, could enhance ASD and ADHD therapies, and may constitute a treatment target for age-related cognitive decline as well. In this context, it's important to note that multiple subpopulations of CD103^+^ immune cells exist that may distinctly impact neurodevelopmental and age-related outcomes. For example, reduction in youth-associated recent thymic emigrant CD103^+^ CD8 T cells, or in immune-priming CD103^+^ dendritic cells (22, 64, 65), may effectively promote neurodevelopmental symptoms of ASD and ADHD, while reduction of age-associated T_RM_ or regulatory T cells (30) may protect against age-related cognitive decline in CD103-deficient animals. Similarly, increasing youth-associated CD103^+^ immune cell levels could potentially prevent or treat ASD and ADHD symptoms, while increasing age-associated immune cells could actually worsen age-related cognitive decline. It is therefore imperative to examine the distinct roles of these CD103^+^ subpopulations in neurodevelopmental and neurodegenerative outcomes, as well as ways to differentially target them.

In summary, we have shown that a single immunological factor promotes sex-disparate ASD- and ADHD-like symptoms with associated neuropathological biomarkers and late-life cognitive protection in mice. This represents a potentially improved model for each disorder, and is the first demonstration that a single immune-based defect can promote both ASD and ADHD behaviors. This is a departure from many other studies of immune activity in ASD and ADHD suggesting hyper-activation of immune cells and responses (7, 66, 67). Our findings also reveal the possibility that cellular immune function may broadly impact neurodevelopment and age-related cognitive decline, and as such may represent a novel target for intervention in these conditions.

## Data Availability Statement

The raw data supporting the conclusions of this article will be made available by the authors, without undue reservation.

## Ethics Statement

The animal study was reviewed and approved by Cedars-Sinai Institutional Animal Care and Use Committee.

## Author Contributions

MJ and AP conducted flow cytometry, Western blots, behavioral assays, and mouse colony management. RC conducted flow cytometry, behavioral assays, and mouse colony management. DI isolated NPCs, designed and performed co-culture studies. LV performed co-cultures and Western blots. NY conducted behavioral assays and mouse colony management. RP designed behavioral assays and ran behavioral core. HS analyzed behavioral assays. KB evaluated the studies' relevance to human conditions. CW designed all studies, analyzed and compiled data, and wrote the manuscript. All authors contributed to the article and approved the submitted version.

## Conflict of Interest

CW was the author of patents PCT/US2016/049598, WO 2017/040594, and PCT/US2019/017879. RC and KB are co-authors on patent PCT/US2019/017879. PCT/US2016/049598, WO 2017/040594 is licensed by Cedars-Sinai Medical Center to T-Neuro Pharma, Inc. CW has received salary and ownership interest in T-Neuro Pharma, Inc. The remaining authors declare that the research was conducted in the absence of any commercial or financial relationships that could be construed as a potential conflict of interest.
