# Supplementary material for: CD103 Deficiency Promotes Autism (ASD) and Attention-Deficit Hyperactivity Disorder (ADHD) Behavioral Spectra and Reduces Age-Related Cognitive Decline
Source: Front Neurol. 2020 Dec 23;11:557269. doi: 10.3389/fneur.2020.557269 (PMC7786306; doi:10.3389/fneur.2020.557269)
Supplement: Supplementary file 1 [file Data_Sheet_1.docx]

Supplementary Material

##
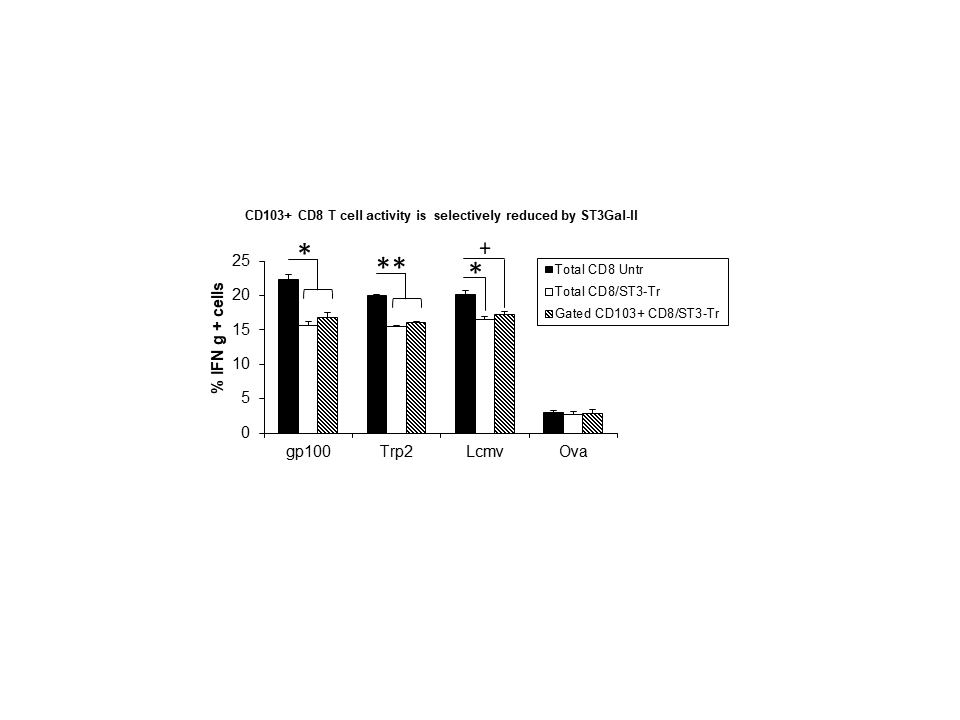


**Supplementary Figure 1.** *CD103^+^ CD8 T cell function is selectively reduced by ST3Gal-II.* Surface de-sialylation is necessary and sufficient for enhanced reactivity to pMHC I stimulation by CD8^+^ T cells. CD8 T cells purified from healthy C57BL/6 mice were stimulated with or without treatment with ST3Gal-II enzyme (ST3-Tr). Stimulation was performed by addition of the indicated self (gp100, Trp-2), endogenous viral (Lcmv) or foreign (OVA) peptide/MHC I tetramers in the presence of 1μg/ml anti-CD28 mAb (Becton-Dickinson) to cultures, followed by incubation at 25^o^C. IFNγ production was determined by combined surface and intracellular flow cytometry (anti-CD3, anti-CD8, anti-CD103, anti-IFNγ) 3 days later. IFNγ reduction in the mixed population, and by CD103^+^ CD8 T cells specifically, was assessed by electronically gated prior to IFNγ assessment. The data show that CD103^+^ cells account for almost all functional reduction in CD8 T cells interacting with endogenous antigens expressed by cells of the central nervous system. ^+^*P* < 0.1, **P* < 0.05, ***P* < 0.01 by 2-sided T-Test in >4 biological replicates (normal distribution/*P* < 0.05 of data confirmed in Shapiro-Wilk test).

Supplementary Methods

*Sialylation and de-sialylation*: Sialic acid was added to surface terminal glycans of CD8 T cells using ST3Gal-II enzyme (Calbiochem #566227) as follows: 1 x 10^6^ CD8 cells per well were plated on a 96 U-bottom well plate in 50 µl ofRMPI/5% FBS. After centrifugation, the plate was placed on ice and enzyme added (5.5 µl CMP-Sialic acid plus 7.54 µl of ST3Gal-II enzyme in a final volume of 50 µl) and cells incubated 30 minutes at 37°C in 5% CO_2_. The plates were then rinsed twice with media and cells used either *in vitro* experiments. PNA staining was first analyzed by flow cytometry before any experiments to ensure that quality of enzyme treatments was correct. *Functional assay*: Mixed CD103^+^ and CD103^-^ CD8 T cells were were purified from mouse spleen by MACS column. Cells were stimulated overnight by the indicated antigen-MHC I tetramers (0.5 µl peptide/MHC I tetramers plus 1 µg anti-CD28 mAb in RPMI-5 in 5% CO2) and expression of IFNγ analyzed on a FACScan II cytometer and Cell Quest software. Duplicate cell aliquots were treated first with ST3Gal-II prior to stimulation.
